# Benzo[a]pyrene stimulates miR-650 expression to promote the pathogenesis of fatty liver disease and hepatocellular carcinoma via SOCS3/JAK/STAT3 cascades

**DOI:** 10.1093/jmcb/mjab052

**Published:** 2021-08-27

**Authors:** Yang Ge, Pengfei Gu, Wenbo Wang, Liyuan Cao, Lulu Zhang, Jingquan Li, Wei Mu, Hui Wang

**Affiliations:** 1 State Key Laboratory of Oncogenes and Related Genes, Center for Single-cell Omics, School of Public Health, Shanghai Jiao Tong University School of Medicine, Shanghai 200025, China; 2 Department of Oncology, Shanghai Tenth People's Hospital, School of Medicine, Tongji University, Shanghai 200072, China; 3 Institute of Military Health Management, Second Military Medical University, Shanghai 200433, China; 4 CAS Key Laboratory of Nutrition, Metabolism and Food Safety, Shanghai Institute of Nutrition and Health, Chinese Academy of Sciences, Shanghai 200031, China

**Keywords:** hepatocellular carcinoma, microRNAs, tumor metastasis, SOCS3, prognostic biomarkers

## Abstract

Modern diets, which often feature high levels of fat and charcoal-grilled meat, contribute to the pathogenesis of obesity and non-alcoholic fatty liver disease (NAFLD), resulting in liver cancer progression. Benzo(a)pyrene (B[a]P) is a common environmental and foodborne pollutant found in smoke and fire-grilled foods, which can have an adverse effect on human health. Hepatocellular carcinoma (HCC) is the fifth leading cause of cancer and the second leading cause of cancer-related deaths worldwide. The epidemiological studies suggest that both environmental risk factors and chronic liver injury including NAFL are important for HCC development, but the precise mechanisms linking eating habits to hepato-carcinogenesis remain unclear. In the present study, we demonstrated that various miRNAs in B[a]P-exposed tumor cells contribute to tumor metastasis, among which miR-650 could be the most potent inducer. Furthermore, we found that the suppressor of cytokine signaling 3 (SOCS3) is directly regulated by miR-650 and its suppression regulates the activation of the Janus kinase/signal transducer and activator of transcription 3 (JAK/STAT3) cascade. Our findings reveal a possible adverse outcome pathway of SOCS3/JAK/STAT3 regulation in B[a]P-induced HCC progress. These results provide a better understanding of the adverse effects of chronic exposure to B[a]P on human health.

## Introduction

Hepatocellular carcinoma (HCC) is the most common type of primary liver cancer (Yang et al., 2019). Large epidemiological studies have reported that an elevated HCC risk is associated with exposure to ubiquitous environmental and food contaminants (Niu et al., 2016), particularly benzo(a)pyrene (B[a]P) intake. Our findings reveal several unhealthy eating patterns, such as tobacco smoke and consumption of grilled foods (Schutte et al., 2009; Souza et al., 2016). B[a]P is a well-known type of carcinogenic polycyclic aromatic hydrocarbons, leading to covalent DNA modifications and dysregulation of gene expression (Ba et al., 2015a). Upon entering the human body, B[a]P is transported by the blood and lymphatic systems to the organs, including the liver and intestine where it would deposit. Once it is taken up by host cells, B[a]P undergoes metabolic activation by generating reactive oxygen species, as well as regulating toxic metabolites that bind to cellular elements, such as DNA damage. Exposure to B[a]P may favor the transition of non-alcoholic fatty liver disease (NAFLD) to liver cancer in obese populations. Furthermore, oral administration of B[a]P leads to various types of gastrointestinal cancer diseases, particularly liver cancer (Chen et al., 2003; Ba et al., 2015b). Although many studies evaluated the mechanism of HCC metastasis (Liu et al., 2014), there are relatively few exploring the association between dietary habits and HCC progression.

MicroRNAs (miRNAs) are regarded as a class of small non-coding RNAs that negatively regulate gene expression primarily through post-transcriptional modifications (Vasuri et al., 2018; Xu et al., 2018). miRNAs are commonly altered in various types of cancer and they have been functionally implicated in tumor pathogenesis, suggesting that they are important modulators of tumorigenesis (Chen et al., 2018). Notably, dysregulation of miRNAs could alter the signaling pathway cascades via targeting their binding regions that block complementary sequences within messenger RNA (mRNA) tails, the 3′-untranslated region (3′-UTR). Translational applications of miRNAs include their use as disease biomarkers, predictors of prognosis, and novel therapies or treatments. The most exciting application enables potentially disease-specific individualized therapeutic targeting. For instance, miR-30a promotes the migration and invasion of liver cancer cells, which indicates the importance of miR-30a in regulating cancer metastasis (Fu et al., 2018). Recently, many studies have screened the upregulated or downregulated miRNAs in HCC and demonstrated that miRNAs play important roles in the metastasis of HCC (Guo et al., 2018; Zhang et al., 2019). However, few studies have evaluated the roles of miRNAs in liver cancer induced by unhealthy eating practices.

In our previous study, we established HCC cell lines by 4-week long-term B[a]P exposure and figured out that chronic B[a]P exposure did not alter cell growth but promoted cell migration and invasion both *in vitro* and *in vivo* (Ba et al., 2015b, 2016). However, the molecular and toxicological mechanisms underpinning the role of B[a]P in tumor progression are still uncertain. To further explore the precise mechanisms in B[a]P-induced HCC metastasis, we used RNA sequencing to screen the previously established HCC cell lines for dysregulated mRNAs and miRNAs after B[a]P exposure. We found that miR-650 was upregulated in B[a]P-treated cells, along with enhanced HCC cell motility. Additionally, we predicted and analyzed the target genes of miR-650. All the predicted genes were analyzed by Gene Ontology (GO) and protein–protein interaction (PPI) network systems. The suppressor of cytokine signaling 3 (SOCS3) was finally verified to be a direct target of miR-650 and responsible for cell motility via regulation of Janus kinase/signal transducer and activator of transcription 3/interleukin-6 (JAK/STAT/IL-6) cascades. Our present findings suggest that miR-650 may be an important diagnostic predictor in B[a]P exposure-correlated liver cancer, which implicates a potential strategy for improving food safety and public health prevention.

## Results

### Long-term B[a]P exposure dysregulates mRNAs and miRNAs in HCC cells

**Figure 1:**
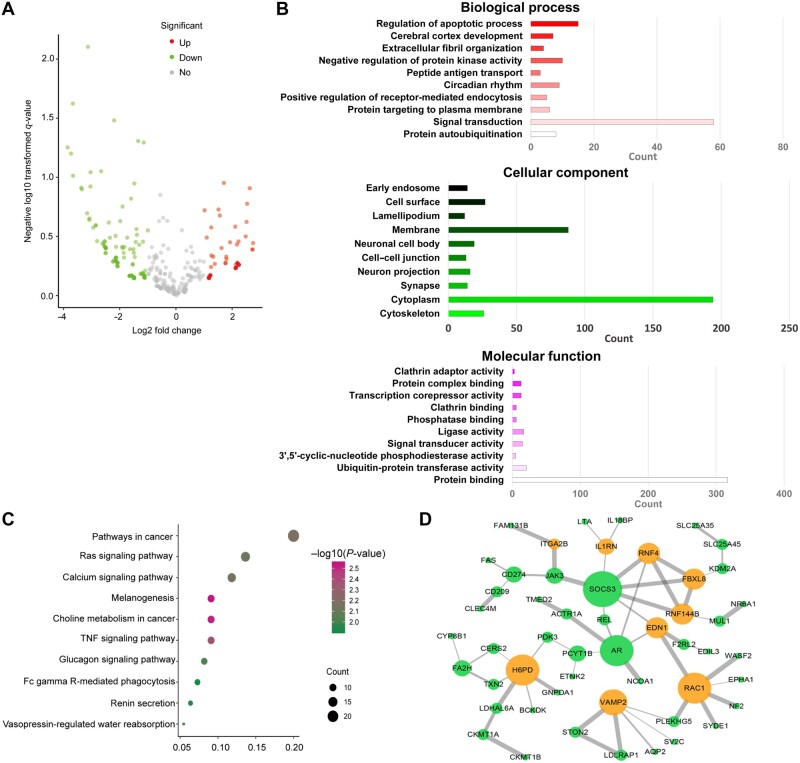
GO, KEGG, and PPI network analyses for functional enrichment in B[a]P-treated HCC cells. RNA sequencing was performed on 7404 cells and 100 nM B[a]P-treated 7404 cells. (**A**) Volcano plot for differentially expressed mRNAs and miRNAs. (**B**) GO analysis of differentially expressed genes in categories of biological process, cellular component, and molecular function. (**C**) KEGG analysis of differentially expressed intersection mRNAs for 10 enriched main pathway terms. (**D**) PPI network organized by String and visualized by cytoscape 3.0, with orange and green spots representing up- and downregulated mRNA-encoded proteins, respectively. Both up- and downregulated mRNAs are significantly changed with FC ≥ 2.0, *P *<* *0.05.

To identify mRNAs and miRNAs that were dysregulated by long-term B[a]P exposure, we performed RNA sequencing to generate mRNA and miRNA profiles of the HCC line 7404 exposed to 100 nM B[a]P (7404-Bap100) and control 7404 cells. The mRNAs and non-coding RNAs were selected by their differential expression, with fold change (FC) ≥2.0, *P *<* *0.05, and false discovery rate (FDR) <0.05. Using these parameters, we identified 348 and 148 mRNAs upregulated and downregulated, respectively. Meanwhile, 76 and 109 miRNAs were upregulated and downregulated, respectively, in 7404-Bap100 cells compared to control cells. The dysregulated genes and miRNAs were analyzed using a Volcano plot, with red points representing upregulation, green points representing downregulation, and gray points representing statistically not significant (Figure 1A). We next analyzed the dysregulated mRNAs in control and B[a]P-exposed cells through GO and Kyoto Encyclopedia of Genes and Genomes (KEGG) analyses. GO analysis revealed the most frequent genes and enriched terms. The profile of annotation consists of biological process, cellular component, and molecular function. Our results showed that in the category of biological process, the most dysregulated mRNAs were associated with signal transduction and regulation of apoptotic process (Figure 1B). In the KEGG database, main pathways were identified, suggesting enriched terms for differentially expressed intersection mRNAs in Ras, TNF, and tumorigenesis cascades (Figure 1C; Supplementary Figure S1). To further investigate the biological functions of mRNAs in B[a]P-exposed HCC cells, we performed a PPI network analysis using the online STRING tool to support the interplay among co-expressing genes. Furthermore, Cytoscape3.0 software was used to visualize and generate predicted PPI networks. As shown in Figure 1D, up- and downregulated mRNA-encoded proteins are coded as orange and green spots, respectively.

### Expression of miRNAs upregulated by B[a]P exposure promotes HCC cell motility

**Figure 2:**
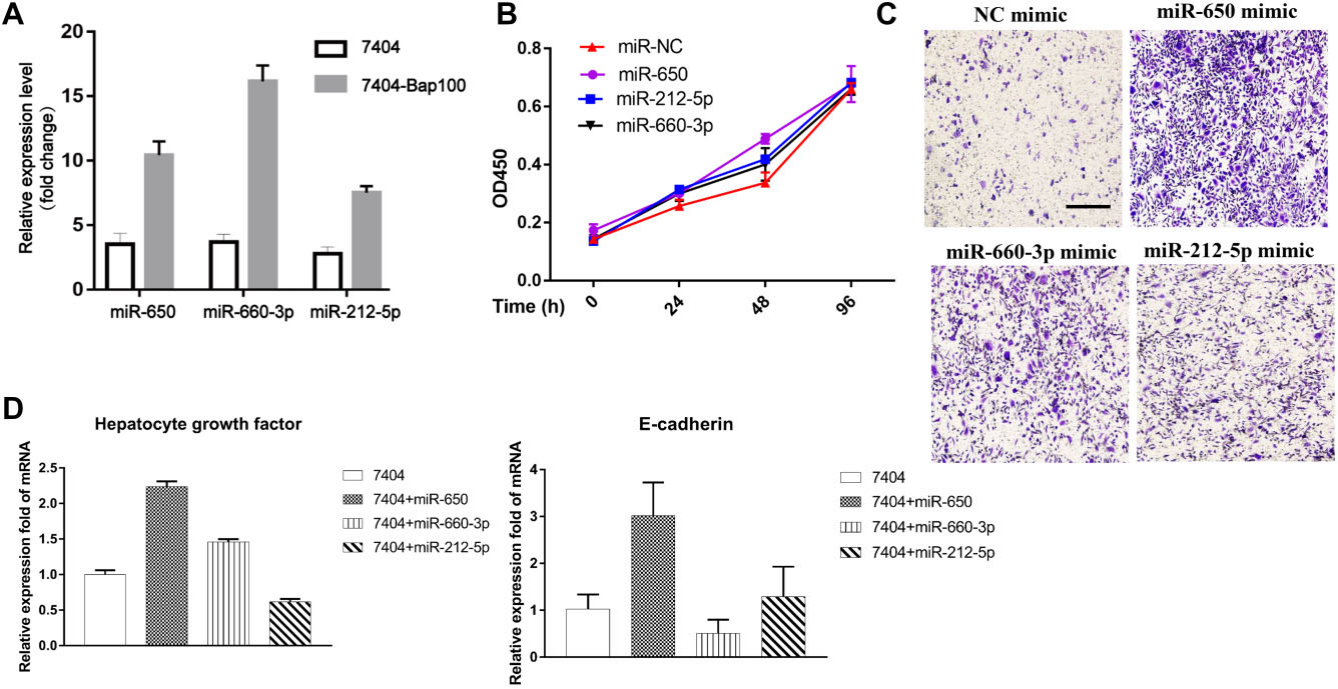
miR-650 is a potent inducer in B[a]P-promoted HCC cell migration. (**A**) Expression levels of miRNAs miR-650, miR-660-3p, and miR-212-5p were determined by qRT-PCR analysis. (**B–D**) 7404 cells were transfected with indicated miRNA mimics or control and analyzed for cell proliferation by CCK-8 assay (**B**), cell migration by transwell assay (**C**), and indicated gene expression by qRT-PCR analysis (**D**).

In our previous study, we found that long-term exposure of B[a]P could not affect the tumor cell growth but significantly facility HCC metastasis (Ba et al., 2015b; Souza et al., 2016). miRNAs are abundant and play important roles in regulating tumor cell proliferation, motility, and apoptosis (Vasuri et al., 2018; Tang et al., 2019). Therefore, we explored the effect of miRNAs on B[a]P-induced HCC cells. To further identify miRNAs specifically induced by B[a]P exposure, we investigated the expression of these candidate miRNAs in 7404 and 7404-Bap100 cells by quantitative real-time polymerase chain reaction (qRT-PCR) analysis (Figure 2A). miR-650, miR-212-5p, and miR-660-3p were highly expressed in B[a]P-treated cells compared to controls. Then, the mimics of these upregulated miRNAs were transfected into 7404 cells, and proliferation and migration assays were performed. The results from the Cell Counting Kit-8 (CCK-8) cell proliferation assay showed little difference between control and miRNA-overexpressing cells, suggesting that B[a]P-induced miRNAs have only a slight effect on tumor cell growth (Figure 2B). Notably, 7404 is regarded as a low-metastatic HCC line, while we had demonstrated that 100 nM B[a]P treatment could significantly promote tumor cell migration. Thus, we next explored the effect of miRNA overexpression on tumor cell motility and found that miR-650 mimic displayed the highest capacity for facilitating cell migration (Figure 2C; Supplementary Figure S2). Meanwhile, we determined that the metastasis-associated genes, such as E-cadherin and hepatocyte growth factor were stimulated in miR-650 mimic-transfected cells (Figure 2D). These results suggested that miR-650 could be a potent inducer in B[a]P-modulated HCC metastasis.

### miR-650 inhibits SOCS3 expression by directly binding to its mRNA

**Figure 3:**
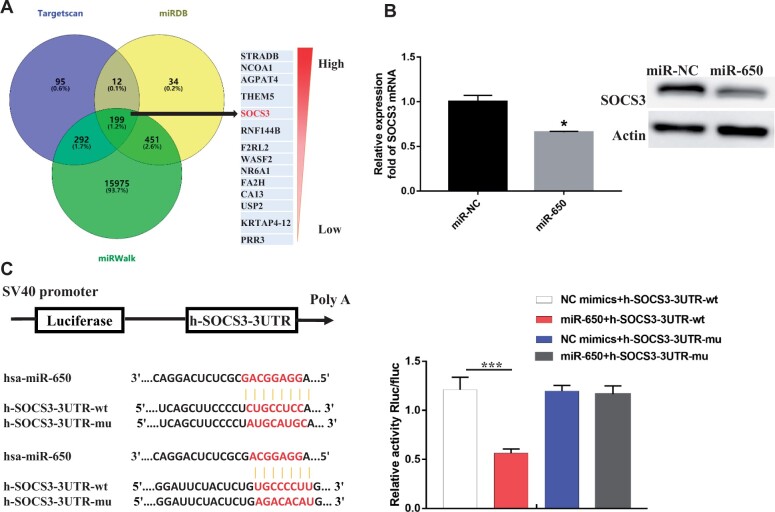
SOCS3 is a direct downstream target of miR-650. (**A**) Target gene prediction of miR-650 with three bioinformatics tools. (**B**) 7404 cells were transfected with miR-650 mimics or control. The transcription and protein expression levels of SOCS3 were determined by qRT-PCR and immunoblotting analyses. (**C**) The luciferase vectors containing wild-type (wt) or mutated (mu) sequences of the binding site between miR-650 and SOCS3 mRNA were constructed. Relative luciferase activities of 7404 cells with the indicated treatments were determined. *P<0.05, ***P<0.001.

The interactions between miR-650 and its target mRNAs were predicted via miRDB, miRWalk, and TargetScan databases, from which a series of common target genes of miR-650 were selected according to the confidence level and predicted binding site scores (Figure 3A). As shown, SOCS3 (Ogata et al., 2006a) was predicted and selected as a potential target of miR-650, which may be responsible for B[a]P-induced HCC metastasis. Interestingly, we also found downregulation of SOCS3 in B[a]P-treated 7404 cells (Figure 1D). Based on that, we explored whether the dysregulated expression of SOCS3 was associated with miR-650 in liver tumor cells (Figure 3B). Briefly, the transcription and protein expression levels of SOCS3 were determined in 7404 cells and miR-650 mimic-transfected cells, respectively. The result showed that miR-650 could significantly inhibit SOCS3 expression (Figure 3B). We further performed a luciferase assay to determine the target site of miR-650. The luciferase vectors containing wild-type or mutant 3′-UTR sequences of SOCS3 mRNA were constructed as shown in Figure 3C. It was obvious that luciferase activity decreased markedly in cells co-transfected with the wild-type binding-site vector in the presence of miR-650. However, cells containing the mutated binding-site vector did not show such repression (Figure 3C). These results reveal that SOCS3 is a direct target gene of miR-650 in B[a]P-treated HCC cells.

### miR-650 promotes tumor cell motility via the SOCS3/JAK/STAT3 axis

**Figure 4:**
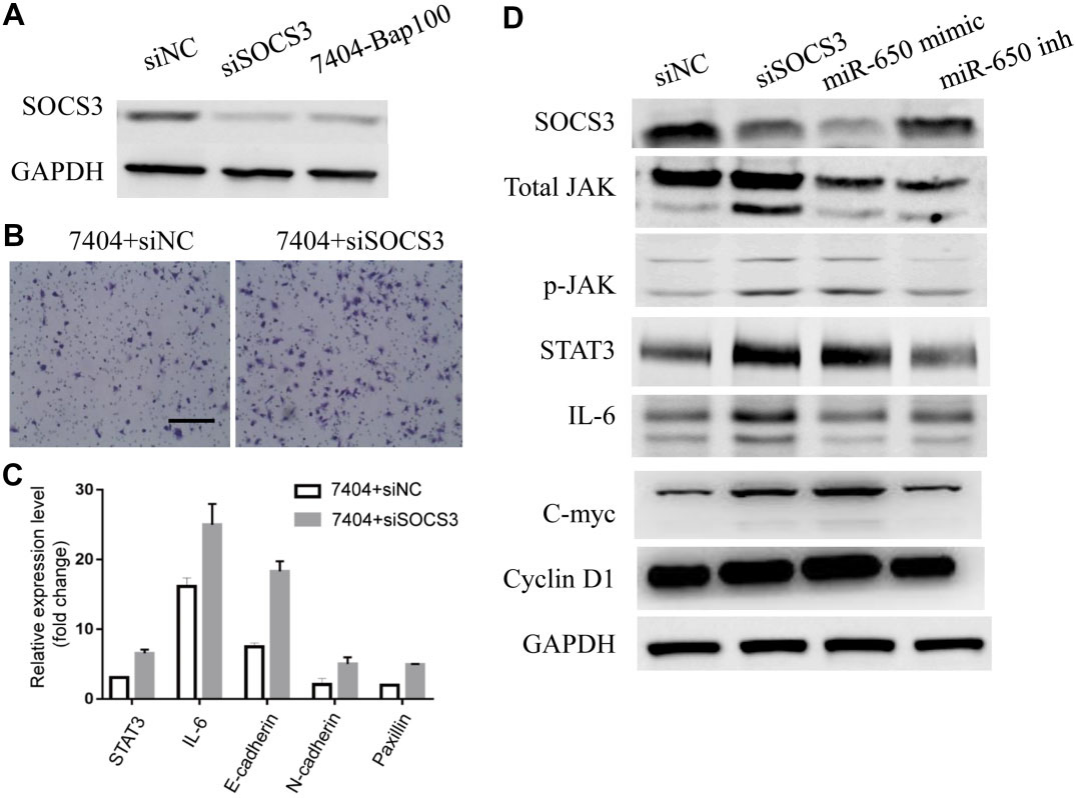
miR-650 promotes tumor cell motility via SOCS3/JAK/STAT3 signaling axis. (**A**) Immunoblotting assay for SOCS3 protein levels in 7404-Bap100, 7404+siSOCS3, and 7404 cells. (**B**) Migration assay of 7404 cells transfected with siRNAs targeting SOCS3 or control. (**C**) qRT-PCR analysis of 7404 cells transfected with siRNAs targeting SOCS3 or control for indicated genes. (**D**) Immunoblotting assays of 7404 cells transfected with siRNAs targeting SOCS3 or control, miR-650 mimic, and miR-650 inhibitor for indicated proteins.

To investigate the effect of SOCS3 inhibition on HCC metastasis, we knocked down SOCS3 expression with small interfering RNAs (siRNAs) in 7404 cells and confirmed the expression levels of SOCS3 in 7404 + control siRNA (siNC), 7404 + siSOCS3, and 7404-Bap100 cells (Figure 4A). Then, cell proliferation and transwell assays were performed in 7404 + siNC and 7404 + siSOCS3 cells. The results revealed very little change in cell proliferation in the absence of SOCS3 (Supplementary Figure S3) but a great promotion on cell motility (Figure 4C), similar to that observed in 7404 cells transfected with miR-650 mimic. Moreover, the expression levels of metastasis-associated and inflammatory genes were examined by qRT-PCR and immunoblotting analyses. As shown in Figure 4C and D, knocking down SOCS3, similar to overexpressing miR-650, enhanced expression levels of oncogenes and pro-inflammatory genes, which were regarded as targets of JAK cascades. Furthermore, both transwell migration and immunoblotting assays were conducted with the miR-650 inhibitor. The miR-650 inhibitor remarkably suppressed the enhanced transwell migration of 7404-Bap100 cells (Supplementary Figure S4), suggesting that miR-650 mediates the B[a]P-promoted HCC cell motility. Additionally, overexpression of miR-650 or downregulation of SOCS3 by siRNA decreased SOCS3 but increased p-JAK and STAT3 expression levels, while opposite results were obtained when treated with the miR-650 inhibitor (Figure 4D). All these data indicate that miR-650 promotes tumor cell motility by mediating SOCS3/JAK/STAT3 signaling activation.

### Downregulation of SOCS3 by B[a]P-induced miR-650a correlates with tumorigenesis

**Figure 5:**
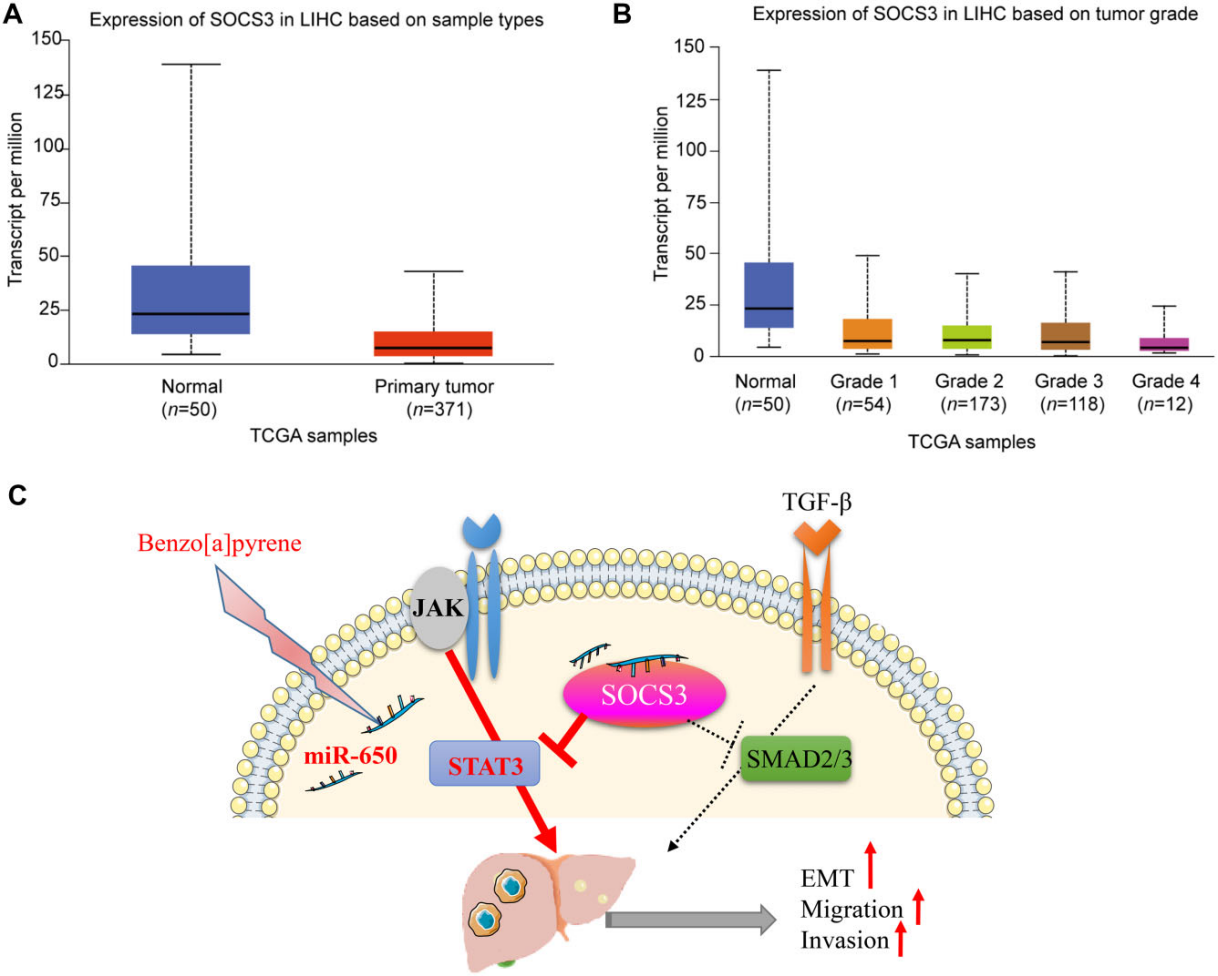
Downregulation of SOCS3 by B[a]P-induced miR-650 may be associated with metastatic progression in liver cancer. (**A**) SOCS3 expression in normal liver tissues and liver cancer tissues. (**B**) SOCS3 expression in normal liver tissues and varying metastasis-grade liver cancer tissues. (**C**) Proposed schematic diagram of B[a]P exposure-stimulated miR-650 mediating SOCS3 suppression and JAK activation to promote tumor cell motility and ultimately metastasis of liver cancer.

We then explored whether the downregulation of SOCS3 by B[a]P exposure-induced miR-650a expression correlates with liver cancer progression in patients. First, SOCS3 expression in liver cancer tissues was much lower than that in normal liver tissues (Figure 5A). Importantly, liver cancer patients suffering from high-grading metastasis showed obviously lower levels of SOCS3 expression (Figure 5B). In addition, although there was no significant difference in overall survival between high- and low-SOCS3 expression groups, lower expression of SOCS3 still responded to a higher percentage of survival (Supplementary Figure S5). In summary, our results suggest a molecular mechanism underlying B[a]P-promoted HCC metastasis that B[a]P-induced miR-650 downregulates SOCS3, thereby activating JAK and STAT3 to promote tumor cell motility (Figure 5C).

## Discussion

HCC is regarded as environmental-related cancer, along with chemical carcinogen components and viral implicated in the multistage process (Tian et al., 2016). Apart from hepatitis B virus or hepatitis C virus infection, obesity-induced NAFLD is a spectrum of chronic liver diseases that range from steatosis to non-alcoholic steatohepatitis and finally to liver cirrhosis or even HCC. Our study explored the effect of direct exposure to environmental pollution (in the form of an unhealthy lifestyle) on cellular changes that destabilize genomic integrity (Jepsen et al., 2012; Younossi et al., 2015, 2016). B[a]P, a polycyclic aromatic hydrocarbon, is one of the ubiquitous environmental pollutants emitted into the surrounding environment via various manners and has been mostly reported in the environment and fried foods as a result of incomplete combustion of organic materials, such as coal, food chain, and cigarette smoke (Conney et al., 1994; Shi et al., 2010). B[a]P is a pro-carcinogen and harmful to human beings, which can be converted into reactive metabolites and contributes to DNA damage (Guo et al., 2015), potentially causing genomic alterations and tumor progression. Although B[a]P has been reported to be associated with increased HCC risk worldwide, relatively little research has been performed to explore the toxicology of long-term exposure to an environmentally low dose of B[a]P in tumor progression. In this study, we first performed RNA sequencing on B[a]P-treated HCC cells and demonstrated that the expression of various mRNAs and miRNAs is dysregulated by B[a]P exposure, which may be associated with HCC metastasis. We observed high expression of miR-650 and decreased expression of SOCS3, an important tumor suppressor, in B[a]P-treated 7404 cells. We then showed that miR-650 directly binds to SOCS3 and may regulate the migratory ability of HCC cells via activation of the JAK/STAT signaling cascade. Furthermore, our analyses suggest that low SOCS3 expression in patients with liver cancer may correlate with tumorigenesis and metastasis, which has important implications for efficient prevention of cancer progression.

Our previous study had demonstrated chronic toxicity of B[a]P with human-derived HCC cell lines that were subjected to long-term B[a]P exposure at environmental-relevant concentrations. Although we have determined the biological effects of B[a]P on cancer metastasis and progression, a better understanding of its underlying adverse outcome pathway is still unclear. Dysregulated expression of miRNAs is commonly observed in HCC, and these miRNAs contribute to tumor development acting as either oncogenes or tumor suppressors (Budhu et al., 2008; Vasuri et al., 2018). Since an individual miRNA can bind to multiple genes and each mRNA also can be targeted by different miRNAs, it is necessary to consider the interaction between miRNA and target gene-associated pathways when investigating the possible functions of miRNAs. The oncogenic role of miR-650 was implicated in several human malignancies (Farooqi et al., 2014; Han et al., 2018; Ningning et al., 2019; Tang et al., 2019). For instance, in prostate cancer, the oncogenic activity of miR-650 is mediated by the downregulation of CSR1 in tumor colony formation (Zuo et al., 2015). Moreover, miR-650 is also overexpressed in anaplastic thyroid carcinoma where it enhances cell migration and invasion through targeting phosphatase 2 catalytic subunit alpha (Orlandella et al., 2019). In this study, we found high expression of miR-650 and suppression of SOCS3 expression in B[a]P-treated HCC cells, which strongly suggested the function of miR-650 as an oncogene by targeting SOCS3 in HCC.

Recently, DNA methylation and reduced expression of the SOCS3 gene in HCC patients have been reported (Ogata et al., 2006a). However, the role of SOCS3 in HCC development *in vivo* has not been clarified. In some types of cancer, the role of SOCS3 seems controversial. Although there are reports of either increased or decreased SOCS3 expression in breast and prostate cancer, SOCS3 functions as a tumor suppressor in most cancer types including gastric cancer, HCC, and colon cancer (Rigby et al., 2007; Kershaw et al., 2013). It has been demonstrated that SOCS3 deletion in the liver resulted in hyperactivation of STAT3 and promoted chemical-induced liver fibrosis (Ogata et al., 2006a). HCC often develops in patients suffering from chronic liver injury to cirrhotic and advanced fibrosis, along with STAT3 and IL-6 activation. Cytokines, including IL-6, activate the JAK signaling pathway, which is utilized by numerous cytokines and is critical for the induction of innate and adaptive immunity (Babon et al., 2012, 2014). Cytokine signaling is strictly regulated by SOCS family proteins. Deletion of the SOCS3 gene in hepatocytes promotes the activation of STAT3, the resistance to apoptosis, and an acceleration of proliferation, resulting in enhanced hepatitis-induced hepatocarcinogenesis (Ogata et al., 2006b). Thus, miR-650-mediated downregulation of SOCS3 expression by B[a]P is generally associated with poor clinical outcome, metastasis, and aggressive phenotype of liver cancer. Our exploration of the adverse outcomes due to B[a]P intake and its role in HCC metastasis have shed light on the toxicological pathway, through which B[a]P acts in HCC progression, and revealed potential targets for public health prevention strategies.

## Methods and materials

### Cell lines and regents

Human HCC cell lines, SMMC-7721 and BEL-7404 (7721 and 7404), were obtained from the Cell Bank of Shanghai Institutes for Biological Sciences, Chinese Academy of Sciences, cultured in RPMI1640 medium supplemented with 10% fetal bovine serum, 100 μg/ml penicillin, and 100 μg/ml streptomycin, and maintained in an incubator with a humidified atmosphere of 5% CO_2_ at 37°C. For B[a]P exposure, 7404 cells were co-cultured with 0.01, 1, and 100 nM B[a]P or 0.1% DMSO for up to 4 weeks. After treatment, B[a]P was withdrawn and the effects of B[a]P on HCC cells were determined. Cell morphology was observed using an inverted microscope. CCK-8 (Dojindo) was used to measure cell growth. miRNA mimics and inhibitors were purchased from Gene Operation. B[a]P, propidium iodide, crystal violet for migration staining, and other chemicals used in this study were purchased from Sigma-Aldrich.

### RNA isolation and miRNA detection

Total RNA from cultured cells, with efficient recovery of small RNAs, was isolated by using the mirVana miRNA Isolation Kit (Qiagen). Detection of the mature form of miR-223 was done by using the mirVana qRT-PCR miRNA Detection Kit and qRT-PCR Primer Sets, according to the manufacturer’s instructions (Qiagen). The U6 small nuclear RNA was used as an internal control.

### Migration and invasion assays

For transwell migration assay, 1 × 10^5^ cells were plated in the top chamber with the non-coated membrane (24-well insert; 8-mm pore size; Corning Costar). For invasion assay, 2 × 10^5^ cells were plated in the top chamber with Matrigel-coated membrane (24-well insert; 8-mm pore size; Corning Costar). In both assays, cells were plated in a medium without serum. Medium supplemented with serum was used as a chemo-attractant in the lower chamber. Cells were incubated for 24 h and the cells that did not migrate or invade through the pores were removed by a cotton swab. Cells on the lower surface of the membrane were fixed with methanol and stained with hematoxylin.

### Luciferase reporter assay

3′-UTR sequences of SOCS3 containing the binding site for miR-650 were cloned into the pMIR-REPORT luciferase construct (Ambion). Cells of 50% confluence in 24-well plates were transfected. Firefly luciferase reporter gene construct (200 ng) and 1 ng of the pRL-SV40 Renilla luciferase construct (for normalization) were co-transfected per well. Luciferase activity was measured after 48 h of transfection by using the Dual-Luciferase Reporter Assay System (Promega).

### Immunoblotting

The total cell lysate was prepared in 1× sodium dodecyl sulfate (SDS) buffer. Proteins were separated by SDS‒polyacrylamide gel electrophoresis and transferred onto polyvinylidene difluoride membranes. Membranes were then blotted with individual antibodies. The bands were visualized by using the enhanced chemiluminescence system (Amersham Pharmacia Biotech) according to the instructions of the manufacturer.

### miRNA mimic transfection

The plasmid vectors encoding miR-650 were constructed and injected into cells. For *in vitro* miRNA inhibition studies, 7404 cells were transfected with the mimic based on the manufacturer’s recommendations. After 48 h of transfection, cells were plated for migration and invasion assays or harvested for luciferase reporter assay.

### Functional enrichment analysis

GO and KEGG functional and pathway enrichment analyses were performed for mRNAs in prognosis-related coexpression RNA network using the Database for Annotation, Visualization, and Integrated Discovery bioinformatics resources (DAVID; https://david-d.ncifcrf.gov/), and *P *<* *0.05 was considered as the cut-off criterion to screen the enriched terms and pathways.

### Establishment of PPI network

To understand the underlying interaction of mRNAs, the STRING website was employed to construct the PPI network, which was visualized by the Cytoscape software v3.6.1.

### Differentially expressed RNA analysis

The analysis and extraction of differentially expressed miRNAs and mRNAs were conducted and analyzed by using the oncomine (www.oncomine.org), networkanalyst (www.networkanalyst.ca), and pathview (www. pathview.uncc.edu) online databases. |log2FC| > 2 and FDR < 0.05 were considered to be significant. The expression network profiles of miRNAs and mRNAs were used for subsequent manipulation.

### SOCS3 siRNA transfection

The expression vector pcDNA-3.1 SOCS3 was constructed by inserting the SOCS3 open-reading frame sequence into the pcDNA-3.1 vector (Invitrogen). pSilencer3.0 (Ambion) was used for the construction of human SOCS3 siRNA vector psi SOCS3 according to the manufacturer’s protocol. The siRNA transfection into cells was carried out with Lipofectamine 2000 (Invitrogen) according to the manufacturer’s protocol.

### Statistical analysis

The value of *P *<* *0.05 was considered as statistically significant and error bars represent the standard error of the mean (SEM). All experiments were repeated 3 times and a representative experiment result was shown with SEM.

## Supplementary material

Supplementary material is available at *Journal of Molecular Cell Biology* online.

## Funding

This work was supported by grants from the National Natural Science Foundation of China (82173543, 81803269, and 81902939), Shanghai Municipality Health Commission (GWV-10.2-YQ17 and 2019Y0150), Innovative Research Team of High-level Local Universities in Shanghai (YG2017QN68), and the Major Science and Technology Innovation Program of Shanghai Municipal Education Commission (2019-01-07-00-01-E00059).

**Conflict of interest:** none declared.

##  

**Author contributions:** W.M., Y.G., and P.G. performed and analyzed experiments. L.C., W.W., and L.Z. performed the experiments and data analysis. W.M. and Y.G. wrote the manuscript. J.L. and H.W. corrected the manuscript draft. All authors approved the manuscript.

## Supplementary Material

mjab052_Supplementary_Data
