# Supplementary material for: Benzo[a]pyrene stimulates miR-650 expression to promote the pathogenesis of fatty liver disease and hepatocellular carcinoma via SOCS3/JAK/STAT3 cascades
Source: J Mol Cell Biol. 2021 Aug 27;13(8):556–64. doi: 10.1093/jmcb/mjab052 (PMC8697348; doi:10.1093/jmcb/mjab052)
Supplement: mjab052_Supplementary_Data [file mjab052_supplementary_data.pdf]

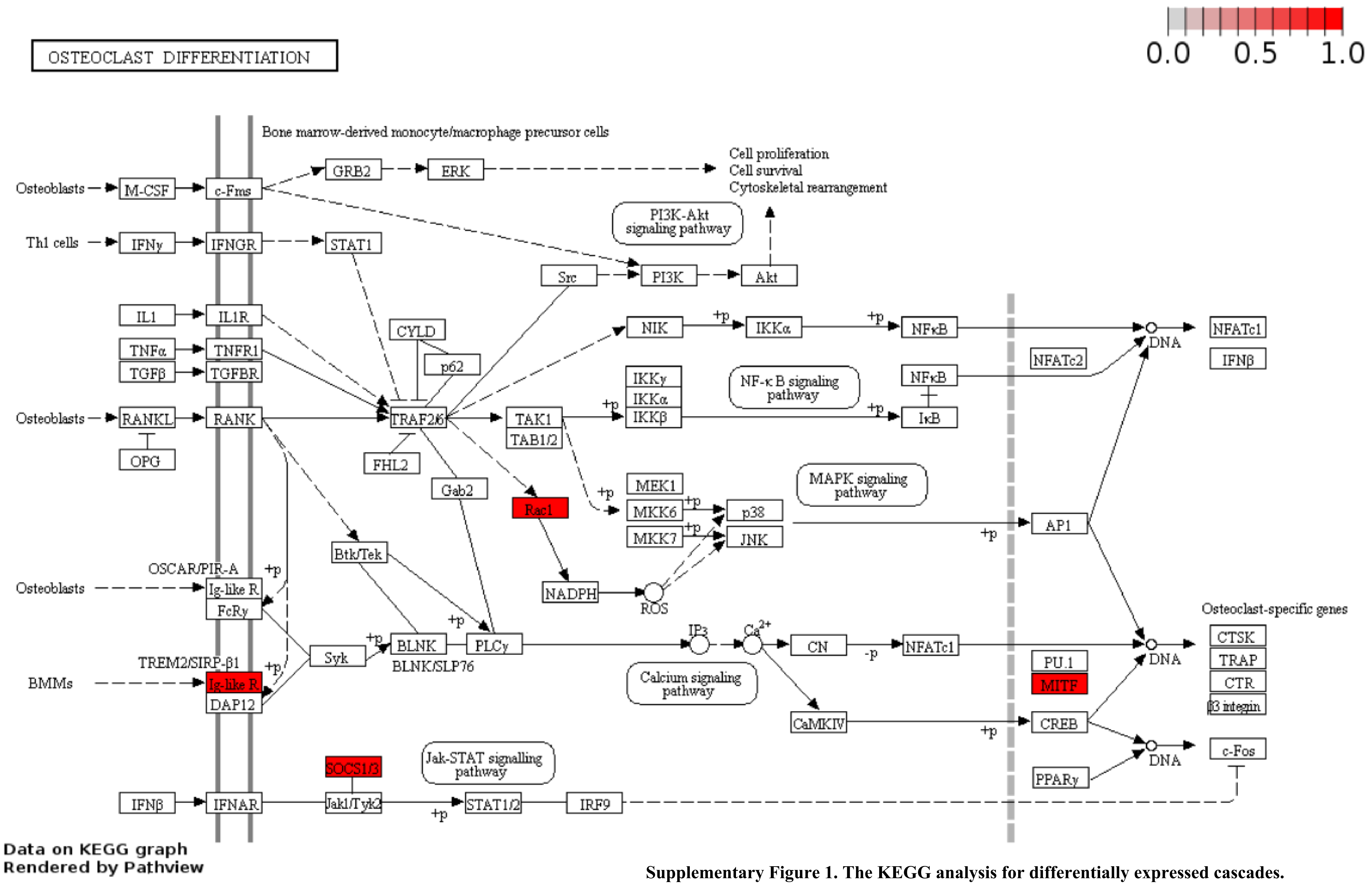

Supplementary Figure 1. The KEGG analysis for differentially expressed cascades.

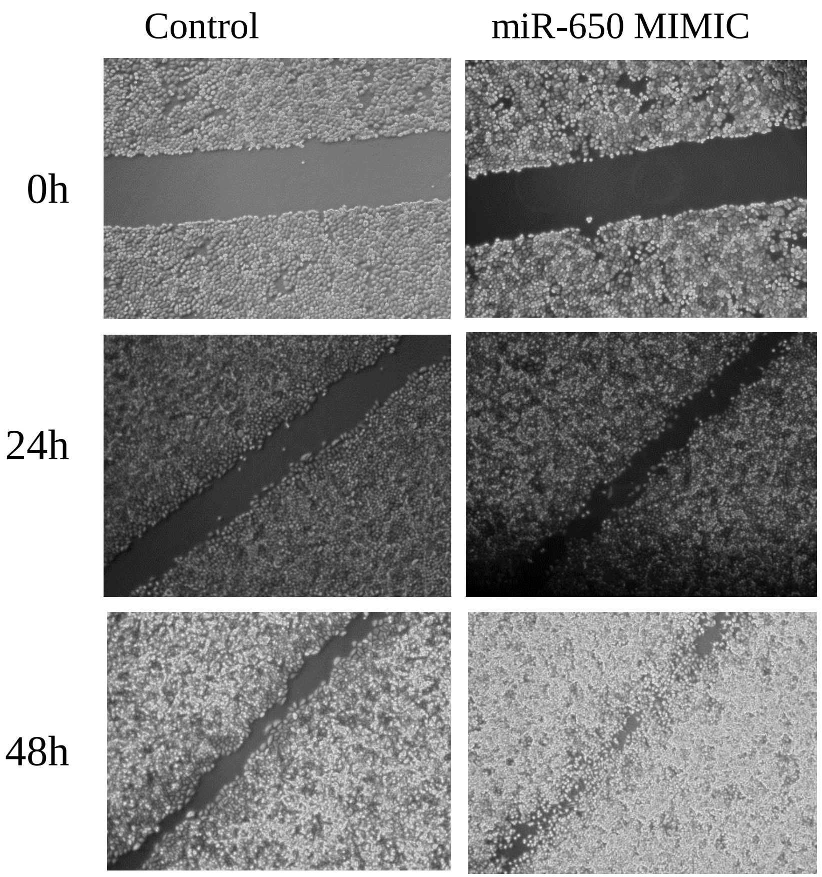

Supplementary Figure 2. The wound healing assay for 7404 and 7404 transfected with miR-650 MIMIC, respectively.

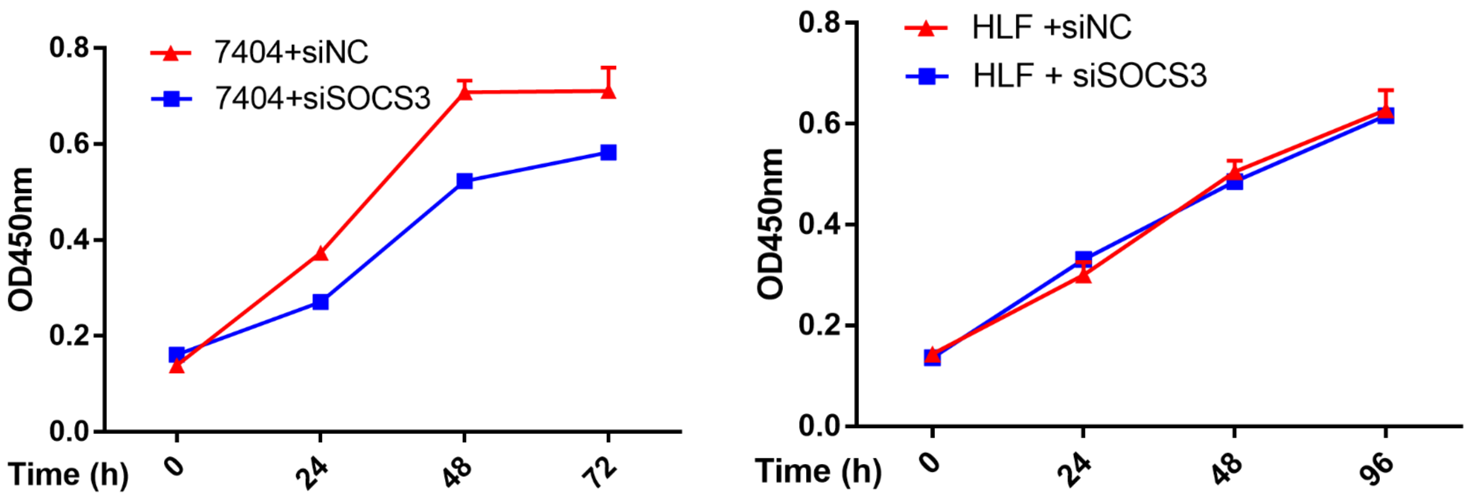

Supplementary Figure 3. 7404 and HLF (human lung fibroblast) cells transfected by siRNA-SOCS3 or siRNA-NC, and cell proliferation was analyzed by CCK-8 assay.

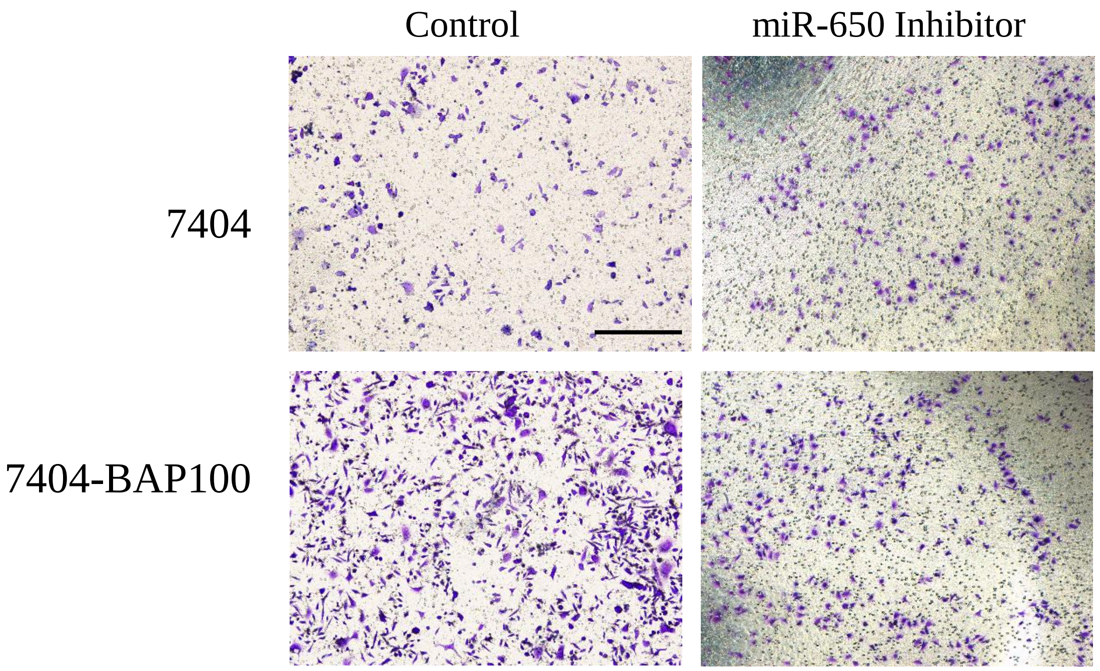

Supplementary Figure 4. Transwell migration assay for 7404 control and 7404 with miRNA-Inhibitor.

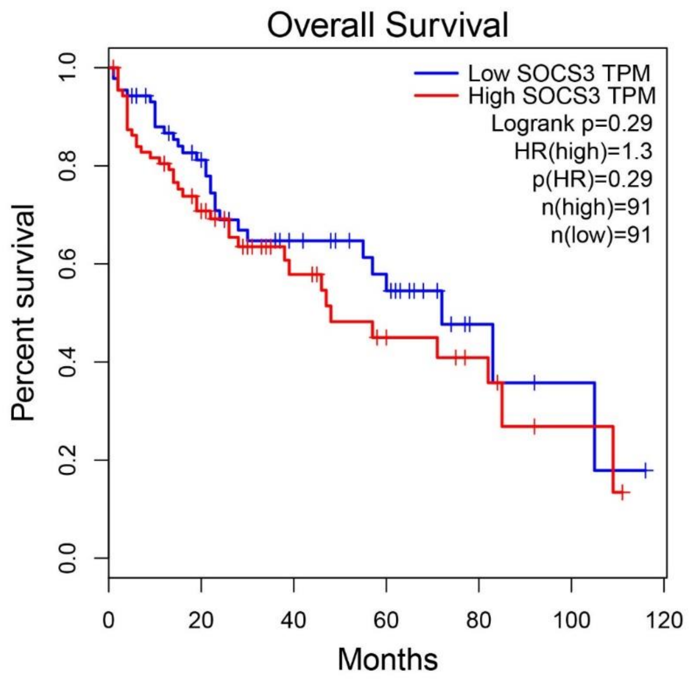

Supplementary Figure 5. Survival data were analyzed by the Kaplan−Meier method and log-rank test.
